# Social and emotional impact of anterior drooling in school-age children and young people with neurodevelopmental disabilities

**DOI:** 10.1007/s00431-024-05714-0

**Published:** 2024-08-15

**Authors:** Lynn B. Orriëns, Lieke G. J. M. van Aarle, Corrie E. Erasmus, Karen van Hulst, Jan J. W. van der Burg

**Affiliations:** 1 Department of Paediatric Neurology, Division of Paediatrics, Donders Institute for Brain, Cognition and Behaviour, Amalia Children’s Hospital, Radboud University Medical Centre, Geert Grooteplein Zuid 10, Nijmegen, the Netherlands; 2 ’s Heeren Loo Zorggroep, Advisium, Amersfoort, the Netherlands; 3 Department of Rehabilitation, Donders Institute for Brain, Cognition and Behaviour, Amalia Children’s Hospital, Radboud University Medical Centre, Nijmegen, the Netherlands; 4 School of Pedagogical and Educational Science, Radboud University Nijmegen, Nijmegen, the Netherlands; 5 Department of Paediatric Rehabilitation, Sint Maartenskliniek, Nijmegen, the Netherlands

**Keywords:** Drooling, Neurodevelopmental disability, Social-emotional development, Impact

## Abstract

**Purpose:**

Anterior drooling is a common comorbidity in children and young people (CYP) with neurodevelopmental disabilities. This study aimed to assess the social and emotional impact of drooling in CYP with a developmental age (DA) of 6 years and older, in whom this impact may differ from those with a lower DA due to their developing sense of self and awareness of their position within social groups.

**Methods:**

Questionnaire data collected for routine clinical care were used to assess parental perceptions of the impact of drooling on (1) social interaction; (2) satisfaction with social interaction, appearance, family relations and life in general and (3) the way CYP expressed feelings on appearance, acceptance by peers and acceptance by adults. Fisher’s exact tests and Mann–Whitney *U* tests were applied to identify associations between clinical characteristics and the social and emotional impact of drooling.

**Results:**

Seventy-nine CYP with an estimated DA ≥ 6 years were included. The majority experienced frequent to constant (83%) and profuse (61%) drooling. Drooling frequently compromised social interaction with peers (49%) and adults (28%), and cognitive abilities were underestimated in 40%. Dissatisfaction with physical appearance (25%) related to drooling was noted. One-fifth of CYP reportedly expressed negative feelings on acceptance by peers related to drooling.

*Conclusions*: These findings underscore the substantial impact of drooling on CYP with a DA of 6 years and older, primarily through avoidance by peers and underestimated cognitive abilities, emphasizing that recognizing and addressing these social-emotional consequences should be integral to clinical care.

**Table:** 

**What is Known:** • *Anterior drooling is common among children and youth with neurodevelopmental disabilities.*
**What is New:** • *There seems to be a heightened prevalence of impaired social interaction with peers and underestimation of cognitive abilities due to drooling among children with a developmental age of at least 6 years compared to previous studies with more heterogeneous populations.* • *The impact of drooling can extend to domains that affect self-esteem, although this may not be fully captured with standardized questions, requiring clinicians to address these consequences in a way that is tailored to the child’s experiences.*

**Supplementary Information:**

The online version contains supplementary material available at 10.1007/s00431-024-05714-0.

## Introduction

While drooling is a natural phenomenon in infants due to the gradual development of oral motor control [1], children generally gain sufficient control over their saliva by the age of 4 [2]. However, persistent difficulties in managing saliva are frequently observed in children and young people (CYP) with neurodevelopmental disabilities. Among CYP with cerebral palsy (CP), the most prevalent motor disability in childhood, chronic anterior drooling affects approximately 44% of all individuals [3]. Addressing these saliva control problems requires an interdisciplinary approach, involving interventions to enhance the self-management of saliva, reduce saliva production or reroute salivary flow [4–6]. These strategies aim to diminish the severity and frequency of drooling, thereby mitigating its impact on CYP and their families [7–9].

The impact of drooling on daily life is known to vary among individuals, but generally encompasses four key domains. First, drooling is often associated with practical consequences (e.g. frequent changing of bibs and clothes; damaged toys, furniture and electronic devices; repeated instructions to swallow), placing an increased burden on caregivers and reducing the independence of CYP [7]. Second, it can have implications for physical health and personal hygiene, resulting in irritated or macerated facial skin, (peri)oral infections, constant damp clothing and malodour [1]. Third, limitations in social interaction may arise due to the effect of drooling on physical appearance and speech intelligibility [1, 7]. As a result, and fourth domain, CYP may be avoided by peers and adults, resulting in difficulties participating in daily activities and negative consequences for their emotional well-being [7], potentially impacting their social-emotional development.

Social-emotional development can be seen as a multifaceted process in which an individual acquires skills, attitudes and behaviours to enable them to understand and manage their emotions, form positive relationships and navigate their social surroundings [10]. Around a developmental age of 6 years, children become more aware of their position within social groups, begin to compare themselves to their peers and start to develop a sense of self and experience feelings of acceptance [11, 12]. However, the physical effects and potential social stigma associated with drooling may contribute to social isolation, reduced opportunities for social engagement and negative remarks from peers, potentially impacting the social-emotional well-being of these children.

Prior research on the impact of drooling in daily life, including its social and emotional consequences, has primarily reported on children with an estimated developmental age below 4 years [13, 14]. However, these findings may not directly apply to CYP with a developmental age of 6 years and older, as self-reflection and feelings of self-esteem become more prominent in this developmental phase. Moreover, CYP of this developmental age may encounter a different social environment with regard to the schools they attend, their interaction with peers beyond the school setting and the social expectations imposed on them [11]. A previous study among children with CP, enrolled in a special education school, who were able to self-report identified that drooling significantly impacted CYP’s interaction with friends and adults [15]. This highlights the necessity to explore the social and emotional impact of drooling in CYP with a developmental age of 6 years and older, which was the primary focus of the current study.

## Methods

### Study design and population

This cross-sectional study included data obtained from questionnaires on the impact of drooling administered as part of routine care in our tertiary care, multidisciplinary saliva control clinic. The study population consisted of CYP (6–25 years old) with a neurodevelopmental disability, who visited the outpatient clinic between May 2000 and May 2023 and for whom an impact questionnaire was completed by parents or caregivers (referred to as ‘parents’ throughout the manuscript). We excluded CYP with an estimated developmental age below 6 years and those with missing data for all items pertaining to social and emotional consequences of drooling in the completed questionnaires. Developmental age was classified into one of three categories (i.e. < 4 years, 4–6 years, ≥ 6 years; reflecting presumed differences in developmental stages regarding self-image, social position, social relationships and abilities to express oneself on these aspects [16]) based on a comprehensive evaluation of the individual’s overall level of functioning in daily life, rather than relying solely on IQ or specific scoring systems. This approach aimed to provide a holistic impression, encompassing domains including but not limited to cognition [17]. Information was integrated from various sources, including tests at rehabilitation centres; (psychological) assessments, the curriculum and offered learning materials at (special education) schools; and parental observations regarding their child’s developmental abilities and self-consciousness [18], ensuring a reflection of the real-world context [8].

Demographic and clinical characteristics of included CYP were obtained from their medical records. This study was conducted in accordance with national and international ethics standards. The original protocol for the assessment of parent-reported questionnaires on the impact of drooling prior to and after interventions was approved by the local research ethics committee in 1999 (file no. 9907–0139). Since all data were collected during routine clinical care, including the questionnaire, no additional informed consent was obtained from parents and/or CYP for the secondary use of this data.

### Outcome measures and definitions

A questionnaire on the impact of drooling on daily life, social interaction and self-esteem was developed at the commencement of our saliva control team, in cooperation with parents of children with saliva control problems [13]. An abbreviated version [19] has been implemented in routine clinical care and was generally administered in all children visiting our saliva control clinic over the past two decades. Face and content validity of the questionnaire were supported by a review of the items by professionals and parents of children with CP and saliva control problems who visited our clinic; it was confirmed by these parents that the questionnaire covers all relevant aspects in which drooling influences daily life. Although no further specific psychometric testing was undertaken, the application of the questionnaire during clinical care and research has demonstrated its correlation with primary measures on drooling severity and its sensitivity to change [8]. Reliability of responses is enhanced by asking respondents to consider only the past month in their answer.

Four different domains are addressed within the questionnaire: (1) the severity of drooling in general, (2) practical consequences and consequences for care, (3) social consequences and (4) emotional consequences (Appendix). Items on the general severity of drooling and its consequences on social and emotional functioning (items 1 and 8 through 17) were selected for the current study.

The social consequences of drooling were addressed through three questions. Parents were asked to indicate whether their child had encountered the following situations: (1) being avoided by other children, (2) being avoided by adults and (3) experiencing underestimation of their developmental age. Subsequently, they were asked to indicate whether or not they believed that drooling played a role in these situations. Both dichotomous answers were combined to assess the impact of drooling on each of these situations.

The emotional consequences domain consisted of two different aspects. First, parental perceptions of their child’s level of satisfaction with social interaction, physical appearance, relationships with family members and life in general were queried. A visual analogue scale (VAS) score was used to represent parental impressions regarding CYP’s level of satisfaction (0 = very dissatisfied, 100 = very satisfied) and the role of drooling within this level of satisfaction (0 = no role at all, 100 = very important role). These continuous scores were categorized into two parts (0–50 and 51–100 for dissatisfied vs satisfied, or drooling plays no important role vs drooling plays an important role, respectively) or three parts (0–32, 33–66, 67–100 for low vs medium vs high satisfaction, or drooling plays no vs moderately important vs very important role, respectively). Second, parental observations regarding their child’s expressions about their physical appearance, feelings of being accepted by adults and feelings of being accepted by other children were addressed.

To quantify drooling severity and/or frequency, the Drooling Quotient (DQ) [20] and the Drooling Severity and Frequency Scale (DSFS) [21] were used.

### Statistical analysis

Data processing and all statistical analyses were conducted with IBM SPSS Statistics (version 27.0; IBM Corp., Armonk, NY, USA). Descriptive statistics were used to describe characteristics of the study population and the social and emotional impact of drooling. Fisher’s Exact tests and Mann–Whitney *U* tests, as applicable, were used to explore differences in the impact on each domain across both sexes (female vs male), chronological age groups (< 13 vs ≥ 13 years old), ambulatory level (ambulant vs non-ambulant), school types (mainstream vs special education schools) and drooling severity (mild to moderate vs severe). Analyses were 2-sided and *p*-values of less than 0.05 were considered statistically significant.

## Results

**Fig. 1:**
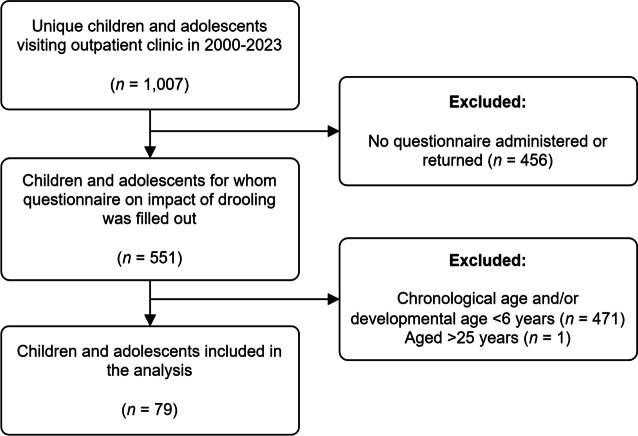
Flowchart representing selection of the study population

Questionnaires were available for 551 CYP who visited the saliva control clinic from May 2000 through May 2023. Of these, 79 individuals (25 females and 54 males) were included in the analyses, as shown in Fig. 1. Characteristics of the study population are presented in Table 1. The median age of the study population was 11.9 years, with a range of 6 years to 23 years (interquartile range [IQR] 6.6 years). The majority of CYP were diagnosed with CP (75%), could walk independently (62%) and were able to communicate verbally (97%), albeit most effectively with familiar individuals (57%). All but three of the CYP attended primary or secondary schools. Of these, 77% were enrolled in special education schools and 12% attended mainstream schools.

**Table 1:** Demographics and clinical characteristics of the study population (*N* = 79)

Characteristics	*n*	(%)
**Age**^a^, years		
6–12	46	(58)
13–18	24	(30)
19–25	9	(11)
**Sex**, female	25	(32)
**Diagnosis**		
Spastic CP	32	(40)
Spastic/dyskinetic CP	17	(22)
Dyskinetic CP	7	(9)
Ataxic CP	2	(3)
Bulbar CP	1	(1)
Other neurodevelopmental disability	20	(25)
**Epilepsy**		
Absent	50	(63)
Controlled	22	(28)
Refractory	7	(9)
**Ambulatory level**, walking	49	(62)
**Dysarthria**^b^		
None	13	(16)
Minimal/mild/moderate	33	(42)
Severe/very severe	33	(42)
**Communication level**^c^ [*n* = 72]		
No effective/incidental verbal communication	2	(3)
Verbal communication for basal needs/with familiar people	41	(57)
Effective verbal communication (despite small problems)	29	(40)
**Feeding method**, oral	72	(91)
**Swallowing on demand** [*n* = 71]		
Normal/slightly aberrant	45	(63)
Impossible/clearly aberrant	26	(37)
**Daytime activity**		
School	76	(96)
Mainstream primary school	8	(11)
Primary special education school	43	(57)
Mainstream secondary school	1	(1)
Secondary special education school	15	(20)
Not specified	9	(12)
Day activity centre or (supervised) work	3	(4)
**Drooling frequency**, frequent or constant^c^ [*n* = 65]	54	(83)
**Drooling severity**, profuse^d^ [*n* = 65]	48	(61)

^a^Chronological age

^b^Classified in accordance with the Paediatric Radboud Dysarthria Assessment at the function level (Ruessink et al., 2022)

^c^Classified in accordance with the activity subscale of the Paediatric Radboud Dysarthria Assessment (Ruessink et al., 2022)

^d^Classified according to the Drooling Severity and Frequency Scale (Thomas-Stonell & Greenberg, 1988)

### Frequency and severity of drooling

Drooling was profuse in the majority (61%) of included CYP and generally (83%) occurred frequently or constantly throughout the day when classified according to the DSFS. Overall, the severity of drooling received a median parent-reported VAS score of 80 (IQR 34.5) out of 100 (0 = no drooling, 100 = very severe drooling). CYP had a median DQ of 12.5 (IQR 40.0). This indicates that new saliva was present beyond the lip margin 12.5% of the time during a 5- or 10-min observation.

### Impact of drooling on social interaction—parental perception

Half of the parents (49%) indicated that they noticed their child being avoided by other children because of drooling (Table 2), whereas being avoided by adults seemed to be a less frequent concern (28%). Additionally, 40% of CYP experienced an underestimation of their cognitive abilities related to drooling according to parents.

**Table 2:** Social consequences of drooling in children and young people with developmental age of 6 years and older observed by parents

Social consequences of drooling observed by parents	*n*	(%)
Avoided by other children [*n* = 77]	46/77	(60)
Avoided by children and drooling plays a role	38/77	(49)
Avoided by familiar or unfamiliar adults [*n* = 74]	28/74	(38)
Avoided by adults and drooling plays a role	21/74	(28)
Cognitive abilities underestimated [*n* = 75]	49/75	(65)
Cognitive abilities underestimated and drooling plays a role	30/75	(40)

Total *n* differs between items due to missing or non-interpretable answers; the number of respondents to each question has been reported in brackets

CYP who were able to walk appeared to have issues with peer rejection more often than CYP who used wheelchairs (61% vs 27%, *p* = 0.005), whereas they were less likely to have their cognitive abilities underestimated due to drooling (29% vs 53%, *p* = 0.034). No evidence was found for a difference in other characteristics.

### Impact of drooling on emotional well-being—parental perception

Overall, dissatisfaction with one’s physical appearance seemed to be the most important emotional consequence of drooling (Table 3). Parents of 17 CYP (25%) indicated their child to be dissatisfied (VAS score 0–50) with their physical appearance and drooling to play a substantial role in this (VAS score 51–100). Moreover, seven CYP (9%) appeared to be dissatisfied with their peer interactions due to drooling, according to parents.

**Table 3:** Emotional consequences of drooling in children and young people with developmental age of 6 years and older observed by parents

Emotional consequences of drooling observed by parents	*n*	(%)
**Satisfaction with peer interaction** [*n* = 74]		
Dissatisfied^a^	21/74	(28)
Dissatisfied and drooling plays a moderately to very important role^b^	7/74	(9)
Highly dissatisfied^c^	9/74	(12)
Highly dissatisfied and drooling plays an important to very important role^d^	3/74	(4)
**Satisfaction with physical appearance** [*n* = 69]		
Dissatisfied^a^	22/69	(32)
Dissatisfied and drooling plays a moderately to very important role^b^	17/69	(25)
Highly dissatisfied^c^	5/69	(7)
Highly dissatisfied and drooling plays an important to very important role^d^	4/69	(6)
**Satisfaction with relationship with family members** [*n* = 74]		
Dissatisfied^a^	8/74	(11)
Dissatisfied and drooling plays a moderately to very important role^b^	2/74	(3)
Highly dissatisfied^c^	3/74	(4)
Highly dissatisfied and drooling plays an important to very important role^d^	1/74	(1)
**Satisfaction with life in general** [*n* = 70]		
Dissatisfied^a^	11/70	(16)
Dissatisfied and drooling plays a moderately to very important role^b^	7/70	(10)
Highly dissatisfied ^c^	4/70	(6)
Highly dissatisfied and drooling plays an important to very important role^d^	1/70	(1)

Total *n* differs between items due to missing or non-interpretable answers; the number of respondents to each question has been reported in brackets

^a^VAS scores 0–50

^b^VAS scores 51–100

^c^VAS scores 0–32

^d^VAS scores 67–100

The extent to which drooling affected satisfaction with physical appearance was significantly greater (*z* =  − 2.02, *p* = 0.044) among girls. Parents reported a median VAS score of 53 (IQR 69) compared to a score of 25 (IQR 52) for boys. Likewise, girls appeared to experience a greater impact of drooling on satisfaction with life in general (median 49; IQR 65 vs median 16; IQR 41; *z* =  − 2.80, *p* = 0.005), according to their parents.

Additionally, CYP attending special education schools seemed to experience a greater influence of drooling on satisfaction with physical appearance (median 32; IQR 57) and family relations (median 14; IQR 33) than CYP enrolled in mainstream schools (median 11; IQR 21; *z* =  − 2.20, *p* = 0.027 and median 3; IQR 3; *z* =  − 2.60, *p* = 0.022, respectively). However, this should be interpreted with caution because of the small sample size in the subgroup of CYP attending mainstream education (*n* = 9).

### Impact of drooling on emotional well-being—parental observations of expressions by CYP

Parents of 20 CYP (28%) indicated that their child exhibited negative reactions related to drooling in one or more areas (Table 4). Most commonly (21%), CYP expressed themselves negatively about their interaction with peers.

**Table 4:** Emotional consequences of drooling expressed by children and young people with developmental age of 6 years and older

Emotional consequences of drooling expressed by CYP	*n*	(%)
Negative feelings about physical appearance [*n* = 71]	17/71	(24)
Negative feelings about physical appearance and drooling plays a role	13/71	(18)
Negative feelings about social acceptance by adults [*n* = 71]	8/71	(11)
Negative feelings about acceptance by adults and drooling plays a role	7/71	(10)
Negative feelings about social acceptance by peers [*n* = 72]	20/72	(28)
Negative feelings about acceptance by peers and drooling plays a role	15/72	(21)

Total *n* differs between items due to missing or non-interpretable answers; the number of respondents to each question has been reported in brackets. Abbreviations: *CYP*, children and young people

Nevertheless, it is noteworthy that a majority of included CYP (51%, 58% and 56%, respectively) did not express themselves either positively or negatively concerning their physical appearance, feeling of acceptance by other children and feeling of acceptance by adults, according to their parents.

## Discussion

This study provides further evidence on the significant impact of drooling on social interaction and emotional well-being of CYP with saliva control problems. Our findings highlight that, according to their parents, CYP with neurodevelopmental disabilities, chronic anterior drooling and a developmental age of 6 years and older frequently encounter social rejection from their peers and often experience an underestimation of their cognitive abilities due to drooling. Additionally, one-fourth of parents reported that their child seemed dissatisfied with how they looked and indicated that drooling played a role in this. A smaller subset of CYP also expressed negative feelings linked to drooling in relation to these aspects.

These findings extend on a previous study by our saliva control team that predominantly delineated the impact of drooling in children with a developmental age below 4 years, with an estimated developmental age of 6 years and older in 11 children [14]. Consistent with our expectations, the current cohort exhibited an even greater frequency of challenges in peer interaction because of drooling (49%) compared to these earlier observations (37%). Additionally, we identified that a greater proportion of CYP with chronic anterior drooling experienced an underestimation of cognitive abilities (40% compared to 19% in the previous cohort). These findings seem to substantiate our initial hypothesis that the developmental age and the social environment of CYP substantially contribute to the degree to which drooling impacts their social opportunities.

With regard to the emotional consequences of drooling, previous studies by our team indicated that only a small group of CYP expressed discontent within important aspects of their lives, including peer interaction, physical appearance, interaction with family members and life in general [8, 14]. Even though median satisfaction scores appeared to be adequate across these domains, our current findings underscore a higher prevalence of emotional consequences among CYP with a developmental age of 6 years and older. Up to 25% of CYP experienced some level of dissatisfaction in these aspects related to drooling, especially regarding their physical appearance, according to parents.

These items on the emotional consequences of drooling in this study relate to elements of the self-esteem domain of the Child Health Questionnaire, a health-related quality of life questionnaire that is widely used to assess the physical, emotional and social well-being of children and adolescents [22]. It is worth considering that the complexity of the emotional impact of drooling may not be fully captured with generic questions like this. Still, our findings highlight the importance for healthcare professionals to acknowledge that self-esteem in CYP with saliva control problems may be affected. In line with our clinical experiences, this appears to be particularly important for the subgroup of CYP at a more advanced developmental stage.

Finally, prior to this study, no definitive conclusions could be reached on the impact of drooling on the self-perception of CYP due to the limited number of children able to express their thoughts on this topic [14]. Even in the current cohort, only half of the included CYP, as reported by their parents, conveyed any type of reaction on their physical appearance or feelings of acceptance. Nonetheless, our findings reveal that 10–21% of CYP voice negative feelings linked to drooling. In fact, when recalculating with the denominator accounting for CYP exhibiting any type of reaction, this percentage rises to a range of 23–47%. The emotional impact of drooling may thus extend even further, though it appears that CYP may not readily articulate their emotions about this, perhaps due to the topic not being a common subject for spontaneous conversation, the question not being raised or awareness of the impact of their emotions on their parents.

Rather than relying solely on standardized questions (e.g. those included in the impact questionnaire used in this study), it would be important to ask CYP how they feel about the effect of drooling on their social and emotional well-being, including friendships, family relationships and having fun—these are deemed significant ‘F-words’ related to participation, linked to the International Classification of Functioning, Disability and Health (ICF) [23]. To accommodate differences in expressive abilities, picture communication symbols may be used to support the discussion. Moreover, the introduction of individualized outcome measures could provide a more comprehensive approach to understanding the social and emotional impact of drooling on the lives of CYP, ensuring that the assessment aligns more accurately with their unique experiences [24].

In recent years, numerous initiatives have focused on optimising the management approach for paediatric drooling, all emphasising the need to consider its impact during assessment and treatment evaluation [4, 9, 25, 26]. Our current findings reinforce these recommendations, confirming the substantial impact of drooling in this subgroup of CYP and underscoring the need for personalised, comprehensive care strategies that address both the physical and social-emotional challenges associated with drooling.

Some limitations of the current study should be considered. First, the developmental age of included CYP was not further specified beyond six years. Consequently, the range in developmental age in our population is not known. Second, developmental age was estimated based on information provided by schools, day care centres, rehabilitation centres and/or parents, and was not objectively assessed upon referral to our saliva control team. Third, we relied on parent-report instead of self-report to evaluate the social-emotional impact of drooling. A cross-sectional study used self-report to assess the social and emotional impact of drooling among 62 children with CP attending special education schools, with questions based on the same questionnaire as used in the current study [15]. Their findings highlight an even more pronounced prevalence of consequences on social interaction and low satisfaction scores across all domains reflecting self-esteem. This may indicate that the perceived impact of drooling in CYP themselves differs from the interpretation of parents. Nevertheless, it is important to note that parents are vital advocates in this patient population [27], and their reporting may offer a broader perspective that takes into account the larger context of their child’s life and justifies the need for care.

To conclude, the current study encompasses an important addition to earlier work. Through a more extensive study population, with a higher developmental age and a varied social environment, our findings emphasize the substantial impact of drooling on the social interaction of CYP. This impact primarily stems from avoidance by peers and an underestimation of cognitive abilities. Importantly, these effects extend into areas that may significantly affect the self-esteem of CYP. Recognizing and addressing these social-emotional consequences should be integral to clinical care, both during the diagnostic workup and the evaluation of treatment outcomes. Acknowledging these consequences not only paves the way for shared decision-making, with a focus on interventions that mitigate the impact of drooling on social-emotional development, but may also foster an increased sense of understanding and enhance trust in healthcare professionals by CYP and their parents.

## Supplementary Information

Below is the link to the electronic supplementary material.

Supplementary file1 (PDF 222 KB)

## Data Availability

The data that support the findings of this study are not publicly available, considering that participants did not provide informed consent for their data to be shared publicly. The data are, however, available from the authors upon reasonable request.
