# Supplementary material for: Social and emotional impact of anterior drooling in school-age children and young people with neurodevelopmental disabilities
Source: Eur J Pediatr. 2024 Aug 15;183(10):4491–8. doi: 10.1007/s00431-024-05714-0 (PMC11413028; doi:10.1007/s00431-024-05714-0)
Supplement: Supplementary file 1 — Supplementary file1 (PDF 222 KB) [file 431_2024_5714_MOESM1_ESM.pdf]

APPENDIX. Questionnaire for parents/caregivers on severity and consequences of drooling

This Appendix serves as a supplement to the paper titled "*Social and emotional impact of anterior drooling in school-age children and young people with neurodevelopmental disabilities*". It provides insight into the items included in the parent-reported questionnaire used to assess the social and emotional impact of drooling in the current study.

<u>Severity of drooling</u>

1. Over the past two weeks, drooling was:

   [--]

   very severe   no drooling

2. For each of the situations mentioned below, indicate whether your child drools (you may select multiple situations). First, indicate whether you feel like a situation is applicable, by selecting 'yes' or 'no'. When you have selected 'yes', indicate the extent of drooling over the past two weeks by choosing between: none (1), mild (2), moderate (3), severe (4), or very severe (5).

	Situation	Applicable?		Extent of drooling				
		Yes	No	1	2	3	4	5
a	Supported sit (e.g. in an adapted chair)							
b	Unsupported sit (e.g. on lap)							
c	Prone position							
d	When your child walks							
e	When your child is active (e.g. sports)							
f	When your child is tired							
g	When your child eats							
h	When your child is focused/concentrated							
i	When your child is relaxed, watching TV							
j	When your child performing a strenuous activity							
k	When your child is excited							

<u>Care aspects and practical consequences of drooling</u>

Could you indicate, as precisely as possible, over the past two weeks:

3. How often is his/her mouth/chin wiped? … times per hour
4. How often is he/she encouraged to swallow? … times per hour
5. How often is his/her bib or scarf changed? … times per day
6. Has drooling resulted in damage to communication aids, communication devices, computer and/or audiovisual devices? yes/no
7. Has drooling resulted in damage to floors and/or furniture? yes/no

Social aspects

A child that drools will sometimes receive reactions from people from his or her environment because of this. When answering the following questions, we would like to know how this has been for your child in the past month.

8. Did you notice your child being avoided by another child? yes/no
   If so, are you under the impression that drooling plays a role in this? yes/no/unsure
9. Did you notice your child being avoided by an adult (familiar or unfamiliar)? yes/no
   If so, are you under the impression that drooling plays a role in this? yes/no/unsure
10. Did you notice that the cognitive ability of your child was underestimated (thought to be lower than it is) by an adult (familiar or unfamiliar) yes/no
   If so, are you under the impression that drooling plays a role in this? yes/no/unsure

Emotional aspects

Parental impression

Questions 11 through 14 concern *your impression* of the extent to which your child is satisfied with their interaction with other children, their appearance, their relationship with family members, and their life in general, as well as the role that drooling plays in this.

11. How satisfied do you think your child has been with his/her interaction with other children, over the past month?

    [--]

    very dissatisfied very satisfied

    In your opinion, to which extent does drooling play a role in this?

    [--]

    no role at all very important role

12. How satisfied do you think your child has been with his/her appearance, over the past month?

    [--]

    very dissatisfied very satisfied

    In your opinion, to which extent does drooling play a role in this?

    [--]

    no role at all very important role

13. How satisfied do you think your child has been with his/her relationship with family members, over the past month?

    [--]

    very dissatisfied   very satisfied

    In your opinion, to which extent does drooling play a role in this?

    [--]

    no role at all   very important role

14. How satisfied do you think your child has been with his/her life in general, over the past month?

    [--]

    very dissatisfied   very satisfied

    In your opinion, to which extent does drooling play a role in this?

    [--]

    no role at all   very important role

Reactions of the child

In the previous paragraph, you were asked about your impressions of the emotional wellbeing of your child. Even though some participants to this study might have limited possibilities to express themselves on this topic, due to their young age and/or communicative limitations, we would like to ask you about the factual reactions of your child in questions 15 through 17.

15. Did you notice your child expressing any overtly positive and/or negative feelings about his/her (physical) appearance?

    ○ no

    ○ yes, in a positive way

    ○ yes, in a negative way → are you under the impression that drooling plays a role? yes/no

16. Did you notice your child expressing any overtly positive and/or negative feelings about the way other adults think about him/her (experience of being socially accepted)?

    ○ no

    ○ yes, in a positive way

    ○ yes, in a negative way → are you under the impression that drooling plays a role? yes/no

17. Did you notice your child expressing any overtly positive and/or negative feelings about the way other children think about him/her (experience of being socially accepted)?

    ○ no

    ○ yes, in a positive way

    ○ yes, in a negative way → are you under the impression that drooling plays a role? yes/no

References

Original publication:

van der Burg JJW, Jongerius PH, van Limbeek J, van Hulst K, Rotteveel JJ. Drooling in children with cerebral palsy: a qualitative method to evaluate parental perceptions of its impact on daily life, social interaction, and self-esteem. *International Journal of Rehabilitation Research*. 2006;29(2):179-82. https://doi.org/10.1097/01.mrr.0000194395.64396.f1

Abbreviated version of the questionnaire:

Kok SE, van der Burg JJW, van Hulst K, Erasmus CE, van den Hoogen FJA. The impact of submandibular duct relocation on drooling and the well-being of children with neurodevelopmental disabilities. *International Journal of Pediatric Otorhinolaryngology*. 2016;88:173-8. https://doi.org/10.1016/j.ijporl.2016.06.043
